# A Wavefield-Domain Method for Refining Residual Timing Errors in Passive-Source Seismic Exploration

**DOI:** 10.3390/s26113567

**Published:** 2026-06-03

**Authors:** Jiawei Song, Guowei Zhu, Qi Li, Yue Zhang

**Affiliations:** 1 College of Geoscience and Surveying Engineering, China University of Mining & Technology, Beijing 100083, China; songjiaweiiiii@163.com (J.S.); 17866562865@163.com (Q.L.); zy69177933@163.com (Y.Z.); 2 SKL for Fine Exploration and Intelligent Development of Coal Resources, China University of Mining & Technology, Beijing 100083, China

**Keywords:** passive-source seismic exploration, signal synchronization, cross-correlation function, residual timing difference, ambient-noise cross-correlation

## Abstract

In passive-source seismic exploration, even after seismic instruments complete unified start-up acquisition and hardware synchronization, long-duration continuous records may still contain small residual timing errors, which in turn broaden cross-correlation peaks and degrade event-location results. To address this problem, this study proposes a wavefield-domain residual timing refinement method. The method uses stable noise windows and controlled artificial events in continuous records as constraints, and performs data-window preprocessing, reference cross-correlation function construction, pairwise residual lag estimation, confidence-weighted multi-station joint fusion, and smoothing-constrained fitting of a continuous correction curve to achieve a posterior refinement of residual timing errors after hardware synchronization. Fractional-delay interpolation is then used for waveform correction. Validation using a 60 min continuous record from a local six-station array shows that the proposed method can serve as an effective supplement to hardware synchronization, suppress residual timing errors, and improve the temporal consistency, waveform stackability, and interpretation reliability of passive-source seismic exploration data.

## 1. Introduction

Passive-source seismic exploration acquires subsurface structural information, local disturbance characteristics, and microseismic activity by continuously recording weak wavefield information generated by natural or engineering disturbances without actively exciting a source [1,2,3,4,5,6,7]. With the development of sensors, embedded acquisition, and distributed networking technologies, miniaturized, multichannel, and low-power acquisition nodes have gradually been applied in mine microseismic monitoring, underground engineering safety assessment, urban shallow-structure exploration, and long-term environmental vibration observation [8,9,10,11,12]. In these applications, data interpretation usually depends not on independent records from a single station, but on inter-station relative arrival times, phase consistency, cross-correlation peaks, and clustering characteristics of repeated events [13,14,15]. Therefore, temporal consistency among multi-station records is a fundamental prerequisite for reliable passive-source seismic exploration results.

Hardware synchronization provides the starting time framework for distributed seismic acquisition, but it does not completely determine the relative timing accuracy required by waveform-based interpretation. After unified start-up acquisition, network timing, hardware triggering, or local clock compensation, small residual offsets may still accumulate during long-duration recording because of field-link fluctuations, local oscillator drift, sampling instability, and environmental noise [10,11]. These residual offsets directly affect the observables used in passive-source interpretation: cross-correlation peaks become broader, coherent phases lose consistency, repeated-event arrivals scatter, and location or stacking results may become unstable when high-frequency components are important [16,17,18]. 

Accordingly, the relevant literature should not be limited to hardware synchronization. A complementary line of research estimates timing errors from the recorded wavefield itself by using ambient-noise cross-correlation, empirical Green’s functions, repeated-event alignment, waveform similarity, and coherent phase relationships [18,19,20,21,22,23,24,25,26,27,28]. This record-driven strategy is attractive because the correction target is defined in the same domain as the subsequent interpretation: if the residual timing error is reduced, the improvement should appear as sharper cross-correlation peaks, more stable inter-station phase relationships, and more repeatable event waveforms rather than only as a clock parameter.

Recent studies further show why this wavefield-domain view is necessary. Nonlinear clock-drift correction for ocean-bottom seismometer networks demonstrates that clock errors may require noise correlation-based tracking and continuous drift curves rather than a single linear correction [29,30,31,32]. Ambient-noise interferometry studies have also shown that selected time-frequency processing and phase-weighted stacking can improve the quality of cross-correlation functions in limited or complex data [33], while seismic-noise matrix imaging illustrates the broader role of noise correlations in high-resolution wavefield monitoring [34]. At the same time, distant storm sources and seasonal noise-source variability can distort noise correlation functions, which means that frequency selection, quality screening, and robust stacking are necessary rather than optional processing details [35].

However, these advances have mainly been developed for regional networks, ocean-bottom seismometer deployments, volcanic monitoring, or long-term ambient-noise studies [29,30,33,34,35,36,37,38,39,40]. Local passive-source seismic exploration has a different operating regime: the array aperture is small, the sampling rate is relatively high, event durations are short, engineering noise can be strong, and acquisition nodes may operate offline. Therefore, the core problem addressed in this study is not to replace the hardware timing system, but to reorganize wavefield-domain timing correction into a complete posterior workflow for local arrays. The proposed method uses waveform similarity in continuous records to construct reference cross-correlation functions, estimate residual lags, fuse multi-station-pair observations, fit a smooth continuous correction curve, and correct the waveform time axis so that the final correction is evaluated directly by waveform coherence, event repeatability, stacking performance, and location stability. The proposed analytical workflow is mainly applicable to localized and small-to-medium-scale engineering applications. In such applications, lateral velocity differences are relatively small; repeatable observed events or stable wavefield components are available; and the influence of propagation paths can be treated as a stable reference within the selected analysis window.

The main contributions of this study are as follows:

(1) A descriptive model of residual timing errors after hardware synchronization in passive-source seismic exploration is established, and the influence of residual timing differences on phase error and waveform consistency is analyzed.

(2) A synchronization-processing workflow based on wavefield-domain residual correction is proposed, including data-window preprocessing, construction of the reference cross-correlation function, residual timing-difference estimation, confidence-based quality control, multi-station-pair fusion, and generation of the continuous time-correction curve.

(3) To address instabilities caused by noise windows, anomalous station pairs, and nonlinear drift in local arrays, robust reweighting and a continuous correction function with smoothing constraints are introduced to improve the stability of the residual-correction results.

To verify the effect of residual timing correction, a validation framework is constructed from three perspectives, namely statistical metrics, waveform-level evidence, and application-level results, so as to demonstrate the improvement of the proposed method in temporal consistency, waveform alignment, and interpretive stability.

The remainder of this paper is organized as follows: Section 2, “Theoretical Basis and Observation Model,” defines the residual timing problem, the phase-error relation, and the wavefield observation assumption; Section 3, “Proposed Residual-Correction Workflow,” converts these assumptions into an executable algorithm; Section 4 validates the method through case studies, parameter analysis, waveform evidence, and application-level results; and Section 5 concludes the paper and discusses future work.

## 2. Theoretical Basis and Observation Model

### 2.1. Distributed Synchronization and Wavefield Timing Calibration

Basic synchronization in distributed seismic acquisition systems generally relies on unified start-up acquisition, network timing, hardware triggering, or local clock compensation. The purpose is to establish a unified starting time reference for different stations so that multinode records can enter the same analysis time framework [10,11]. This type of synchronization belongs to hardware system-level time control and mainly addresses initial offsets and large-scale clock drift. For engineering acquisition systems, hardware synchronization constrains records from different stations to a range in which they can be compared, cross-correlated, and stacked [16,17,18,19,20,21].

Wavefield timing-calibration methods treat residual timing errors as parameters that can be inverted from observational data. For the same event or a stable noise-statistics window, records from different stations should exhibit similar waveform structures or stable cross-correlation responses [18,19,20,21]. When a residual timing shift exists between stations, the cross-correlation peak is displaced relative to the reference function. Therefore, residual timing differences can be estimated by constructing a stable reference cross-correlation function (CCF) and measuring the offset of cross-correlation functions from different time periods relative to this reference [23,36,37].

### 2.2. Residual Synchronization Errors and Phase Errors

After hardware synchronization, residual timing errors may still remain between station pairs. The inter-station timing offset is therefore decomposed into a hardware-synchronization component and a wavefield-domain residual component as follows:

(1) $$\Delta t_{ij}\left(n\right)=\Delta t_{coarse,ij}\left(n\right)+\delta_{ij}\left(n\right)$$

In Equation (1), $\Delta t_{ij}(n)$ denotes the total inter-station timing offset between stations i and j in the nth discrete analysis unit; in the later algorithmic notation, the same discrete unit is denoted by k when it represents a time window or a repeated-event window. $\Delta t_{coarse,ij}(n)$ is the timing component remaining after hardware synchronization, and $\delta_{ij}(n)$ is the wavefield-domain residual term estimated from the observed seismic records. All three quantities have units of time, expressed in seconds or microseconds. Under the adopted sign convention, a positive pairwise residual lag means that the waveform recorded at station i lags behind that at station j. This residual timing difference is then converted into the phase-error relation in Equation (2).

(2) $$\phi_{ij}\left(f,n\right)=2\pi f\;\delta_{ij}\left(n\right)$$

In Equation (2), $\phi_{ij}(f,n)$ denotes the phase error induced by the residual timing difference for the station pair (i, j) at signal frequency f and analysis unit n. The frequency f is measured in hertz, and $\delta_{ij}(n)$ is measured in seconds; therefore $\phi_{ij}(f,n)$ is expressed in radians and can be converted to degrees by multiplying by 180/π. The sign of the phase error follows the residual-lag convention defined after Equation (1). This relationship shows that residual timing differences are amplified at high frequencies, which motivates the subsequent estimation and correction of the residual timing term.

### 2.3. Wavefield Observation and the Cross-Correlation Assumption

For signal sequences associated with the same passive-source event or the same noise field, the observed record at a station is modeled as a shifted and noisy version of a common waveform:

(3) $$x_{i}\left(t\right)=a_{i}\;s\left(\cdot \right)\left(t-\tau_{i}-\delta_{i}\left(t\right)\right)+n_{i}\left(t\right)$$

In Equation (3), $x_{i}(t)$ is the observed waveform at station i as a function of continuous time t in seconds; $a_{i}$ is a dimensionless amplitude-scaling coefficient; s(t) is the ideal reference waveform or common source response; $\tau_{i}$ is the physical propagation arrival time determined by source location, station geometry, and subsurface velocity; $\delta_{i}(t)$ is the station-specific synchronization perturbation with units of time; and $n_{i}(t)$ represents environmental noise, instrumental noise, and other interference terms in the same amplitude unit as $x_{i}(t)$. A positive $\delta_{i}(t)$ denotes a delayed station-i record relative to the adopted reference response. According to this model, the propagation-path contribution is treated as a stable reference shift, whereas the synchronization-error term appears as an additional offset of the cross-correlation peak and is used to invert the residual timing difference.

This observation model does not require a globally homogeneous velocity model. The key requirement is separability: for the selected event family or stable noise window, propagation-path effects should remain sufficiently stable so that time-varying residual offsets can be estimated from the displacement of the cross-correlation peak. If strong lateral velocity variations, changing source paths, or time-varying medium properties dominate the observed arrival-time differences, the estimated lag may mix synchronization error with propagation effects. Such cases should be excluded through window selection and quality control, or interpreted with additional velocity-structure constraints.

In summary, Section 2 defines the physical and mathematical basis of the problem rather than the processing procedure itself. The residual timing term is formulated as an observable wavefield-domain perturbation, and the cross-correlation assumption explains why this perturbation can be estimated from waveform similarity. Section 3 therefore focuses on the operational workflow that turns these assumptions into a practical residual-correction algorithm.

## 3. Proposed Residual-Correction Workflow

Building on the theoretical basis in Section 2, Section 3 converts the residual timing-error model into an operational workflow. The workflow contains six connected stages: data-window selection and preprocessing, reference cross-correlation function construction, residual timing-difference estimation and interpolation, multi-station joint correction, construction of a continuous time-correction curve, and waveform correction.

The role of hardware synchronization in this workflow is limited to providing an initial common time frame and constraining the residual-lag search range. The residual correction itself is estimated from the observed wavefield, so the implementation is organized around cross-correlation stability, quality screening, confidence weighting, robust multi-station fusion, and continuous curve fitting.

This organization clarifies the boundary between Section 2 and Section 3. Section 2 defines the residual timing-error model and the cross-correlation assumption; Section 3 describes how these assumptions are implemented as a reproducible residual-correction workflow, from window preparation to final waveform time-axis correction.

### 3.1. Data-Window Selection and Preprocessing

To balance time resolution and cross-correlation stability, the raw record is first segmented into fixed-length data windows. In this study, “local” refers to the small-to-intermediate engineering-scale array used in the field test, namely six acquisition stations with 1 m inter-station spacing and a nominal aperture on the order of 12 m, and “high sps” refers to the sampling rate of 1000 samples per second (1000 sps, equivalent to 1000 Hz). In the default configuration, the data-window length is 20 s with 50% overlap and the processing band is 5–80 Hz; the 20 s window contains enough coherent wavefield samples for stable cross-correlation at 1000 Hz while preserving sufficient temporal resolution for tracking residual timing changes. Within each window, mean removal; detrending; despiking, namely abnormal impulse removal; band-pass filtering; amplitude normalization; and spectral whitening when necessary are applied before cross-correlation. For repeated calibration events, the analysis window is selected around the main arrival; for ambient-noise records, windows affected by strong transient interference or instrument saturation are avoided as much as possible.

These preprocessing values are not intended as universal constants. For practical deployment, the selected passband should cover the dominant coherent energy of the target wavefield while excluding narrow-band engineering interference and very-low-frequency drift. In this study, the default preprocessing further uses a 1.0 s RMS normalization window, a clipping threshold of four times the median short-window amplitude, and a CCF confidence threshold of 0.65. If the source spectrum, sampling rate, or noise field changes markedly, the passband and screening thresholds should be rechecked using the same peak coefficient, residual delay, and valid-window proportion metrics used in Section 4.2.

To avoid excessive disturbance of the cross-correlation result by abrupt amplitude changes, a combined strategy of short-window root-mean-square amplitude normalization and extreme-value clipping is adopted. Windows that clearly deviate from the overall statistical distribution are not directly included in stacking or subsequent estimation, but are removed during the quality-control stage.

Because local passive-source records often contain background noise, engineering disturbances, and instrumental transient responses simultaneously, a two-threshold screening strategy is further adopted during preprocessing. Saturated segments are first removed according to short-window energy, and interference windows with abnormally concentrated frequency bands are then excluded according to spectral flatness. This treatment improves the effectiveness of the valid windows used to construct the reference function and reduces the systematic contamination of residual timing-difference estimation by a small number of abnormal windows.

(4) $$x_{i}^{\left(m\right)}\left(t\right)=\frac{B\left\{D\left[x_{i}\left(t\right)\right]\right\}}{\sqrt{\frac{1}{T_{w}}\int_{0}^{T_{w}}{\left[B\left\{D\left[x_{i}\left(t\right)\right]\right\}\right]}^{2}\;dt}}$$

In Equation (4), ${\overset{\sim }{x}}_{i}^{m}(t)$ denotes the preprocessed and normalized waveform of station i in the mth analysis window, where t is the local time within the window. D(·) denotes detrending and B(·) denotes band-pass filtering; if written explicitly, the lower and upper cutoff frequencies are denoted by $f_{l}$ and $f_{h}$ in hertz. The denominator represents energy normalization over the window length $T_{w}$, so the normalized waveform is dimensionless and is less sensitive to amplitude differences among stations. Extreme-value clipping, amplitude normalization, and spectral whitening are preprocessing operations applied in the same window when required by data quality. The resulting normalized signal is then used to calculate the short-window cross-correlation function in Equation (5). Here, $T_{w}$ denotes the data-window length used for the energy normalization in Equation (4); its default value is 20 s in this study unless otherwise stated.

(5) $$C_{ij}^{m}(\tau )=\frac{\int_{0}^{T}x_{i}^{m}(t)x_{j}^{m}(t+\tau )dt}{\sqrt{\int_{0}^{T}{x_{i}^{m}(t)}^{2}dt}\sqrt{\int_{0}^{T}{x_{j}^{m}(t)}^{2}dt}}$$

In Equation (5), $C_{ij}^{m}(\tau )$ is the normalized short-window cross-correlation function between the preprocessed records of stations i and j in the mth analysis window. The variable τ is the correlation lag in seconds, and $T_{w}$ is the analysis-window length. Because the numerator is normalized by the waveform energies of both stations, $C_{ij}^{m}(\tau )$ is dimensionless, and its peak position mainly reflects relative waveform alignment rather than amplitude contrast.

### 3.2. Construction of the Reference Cross-Correlation Function

For each station pair and each component combination, the preprocessed short-window cross-correlation functions are grouped either by time window or by repeatable event before stacking. Time-window stacking is used when the stable background wavefield dominates the record, whereas event-based stacking is used when controlled or repeatable events provide clearer and more coherent arrivals. In both cases, stacking is used to improve the signal-to-noise ratio and peak stability of the reference cross-correlation function, rather than to change the mathematical form of the residual-correction model.

Therefore, the choice between time-window stacking and event-based stacking mainly affects the sensitivity of the lag estimate, including peak sharpness, valid-window count, and robustness to non-stationary noise. Once a valid residual lag is extracted, the subsequent steps are identical: the pairwise lag estimates are quality weighted, fused across station pairs, fitted as a continuous correction curve, and applied to the waveform time axis. Thus, the stacking choice does not define a different final correction algorithm; it only determines which observations provide the most stable constraints for the same correction framework.

The initial reference is not assumed to be an exact standard. It is constructed from quality-screened windows after hardware synchronization has already constrained the possible residual offset to a narrow search range. Its role is therefore limited to providing a robust temporary template for first-round lag estimation. Windows with broad, nonunique, or low-confidence peaks are excluded from direct residual inversion or receive low weights, so an unstable local window is prevented from dominating the reference.

After the first-round lag estimates are obtained, the individual cross-correlation functions are realigned and restacked to form the corrected reference function. The corrected reference, rather than the initial reference, is used as the baseline for subsequent residual timing-difference measurement. This design reduces peak-main-lobe broadening caused by residual offsets in the initial stack and removes the apparent contradiction between using an imperfect initial reference and requiring an accurate final reference.

For all cross-correlation windows that pass quality screening, the reference cross-correlation function is constructed by weighted stacking as follows:

(6) $$C_{ij}^{ref}(\tau )=\frac{\sum_{k=1}^{K}\alpha_{ij}^{k}C_{ij}^{k}(\tau +\tau_{ij,0}^{k})}{\sum_{k=1}^{K}\alpha_{ij}^{k}}$$

In Equation (6), $C_{ij}^{ref}(\tau )$ is the reference cross-correlation function for station pair (i, j). K is the number of valid analysis windows included in the stack, $\alpha_{ij}^{k}$ is the dimensionless quality weight of the kth window, and $\tau_{ij,0}^{k}$ is the preliminary alignment shift, with units of time, applied during the first-round reference construction. The reference function provides the baseline against which later residual lags are measured, and the sign of $\tau_{ij,0}^{k}$ follows the same lag convention as the pairwise residual estimate.

### 3.3. Residual Time-Difference Estimation and Interpolation

Residual lag estimation compares each current cross-correlation function with the reference function. A sample-level peak search is first performed, followed by sub-sample interpolation within the peak neighborhood.

For each station pair, residual timing-difference estimation records not only the peak position, but also the peak correlation coefficient, half-peak width, and peak symmetry. The purpose is to distinguish, during subsequent quality control, between results with a stable peak position but poor peak shape and results with both a clear and stable peak, thereby avoiding unstable estimates caused by relying solely on peak position.

If the cross-correlation function in the current window does not satisfy peak uniqueness, or if the peak shape is obviously broadened or the half-peak width is abnormally large, that window can still participate in reference stacking but is not directly used for residual timing-difference inversion. This is because cross-correlation stacking can tolerate a certain amount of noise, whereas time-shift measurement is more sensitive to peak morphology.

For each time or event unit, the sample-level lag is obtained by maximizing the normalized similarity between the current cross-correlation function and the reference function:

(7) $$\tau_{ij}^{\left(k\right)}=arg\underset{\tau }{max}\left\{\frac{\int C_{ij}^{\left(k\right)}\left(\xi \right)\;C_{ij}^{ref}\left(\xi +\tau \right)\;d\xi }{\sqrt{\int {\left[C_{ij}^{\left(k\right)}\left(\xi \right)\right]}^{2}d\xi \;\cdot \;\int {\left[C_{ij}^{ref}\left(\xi \right)\right]}^{2}d\xi }}\right\}$$

In Equation (7), $\tau_{ij}^{k}$ denotes the sample-level residual lag estimate for station pair (i, j) in the kth time or event unit. The integration variable ξ is a dummy lag variable used when matching the current cross-correlation function with the reference cross-correlation function, and the denominator normalizes the similarity measure. The estimated lag is the shift that maximizes this normalized similarity and has units of time; a positive value is interpreted according to the convention that station i lags station j before conversion to a station-specific correction.

To avoid the limitation of the sampling interval on estimation accuracy, parabolic interpolation is performed within the peak neighborhood as follows:

(8) $$\tau_{ij,sub}^{\left(k\right)}=\tau_{ij}^{\left(k\right)}+\frac{\Delta \tau }{2}\cdot \frac{R_{-1}-R_{+1}}{R_{-1}-2R_{0}+R_{+1}}$$

In Equation (8), $R_{0}$ is the normalized similarity at the discrete maximum, whereas $R_{-1}$ and $R_{+1}$ are the similarity values at the left and right neighboring lag samples. Δτ is the lag-sample interval, equivalent to the sampling interval $\Delta t_{s}$ after preprocessing. The interpolation term gives a sub-sample correction to $\tau_{ij}^{k}$, and $\tau_{ij,sub}^{k}$ is the final sub-sample residual-lag estimate in seconds or microseconds.

### 3.4. Multi-Station Joint Correction

To suppress unstable estimates caused by noise windows and anomalous station pairs, each pairwise residual-lag estimate is assigned a confidence weight based on peak coefficient, half-peak width, peak symmetry, and local signal-to-noise ratio. Only estimates exceeding a prescribed quality threshold are retained for joint correction.

In the joint-correction stage, a stability constraint for reference stations is further introduced. If a reference station persistently exhibits low correlation peaks or broad-peak characteristics over multiple time periods, its constraining weight on other stations is automatically reduced to prevent locally unstable stations from dominating the array residual-correction result. Considering that some residual timing-difference estimates may still deviate from the overall trend in local time periods even after passing the initial threshold, a residual-based reweighting step is added after the weighted average. The idea is similar to robust regression: the residual of each station pair is first calculated from the first-round fusion result, and then the influence of anomalous observations is weakened according to the residual magnitude. For passive-source records with limited array scale and noticeable fluctuations in local link quality, this step can markedly reduce the dragging effect of a single abnormal station pair on the final curve.

Before multi-station fusion, the station-pair observations are related to station-specific correction quantities as follows:

(9) $$\tau_{ij}^{\left(k\right)}=\delta_{j}\left(k\right)-\delta_{i}\left(k\right)+\varepsilon_{ij}^{\left(k\right)}$$

In Equation (9), $\tau_{ij}^{k}$ is the observed pairwise residual timing difference between stations i and j in the kth unit, while $\delta_{i}^{k}$ and $\delta_{j}^{k}$ are the corresponding single-station correction quantities, all with units of time. $\varepsilon_{ij}^{k}$ represents measurement noise or modeling error. This equation links pairwise lag observations to station-specific corrections; before the weighted fusion in Equation (11), each pairwise lag is converted to the correction direction of the target station. With the sign convention used here, a positive residual lag indicates that station i is delayed relative to station j, whereas a positive station correction is applied to advance the delayed record during waveform time-axis correction.

Because the peak sharpness, signal-to-noise ratio, peak symmetry, and peak width of different cross-correlation windows are not identical, these quality factors are combined into a comprehensive confidence weight:

(10) $$w_{ij}^{\left(k\right)}=q_{peak,ij}^{\left(k\right)}\;q_{snr,ij}^{\left(k\right)}\;q_{sym,ij}^{\left(k\right)}\;q_{width,ij}^{\left(k\right)}$$

In Equation (10), $w_{ij}^{k}$ is the comprehensive confidence weight of station pair (i, j) in the kth unit. The factors $q_{peak,ij}^{k}$, $q_{snr,ij}^{k}$, $q_{sym,ij}^{k}$, and $q_{width,ij}^{k}$ quantify peak sharpness, local signal-to-noise ratio, peak symmetry, and peak width, respectively. These factors are dimensionless reliability indicators, normally bounded between zero and one, so a low value of any factor reduces the contribution of the corresponding station pair to the joint correction.

After valid station-pair observations are obtained, the station-specific correction is estimated by weighted averaging over the valid reference-station set:

(11) $$\delta_{i}\left(k\right)=\frac{\sum_{j\in \Omega_{i}}w_{ij}^{\left(k\right)}\;\tau_{ij}^{\left(k\right)}}{\sum_{j\in \Omega_{i}}w_{ij}^{\left(k\right)}}$$

In Equation (11), $\delta_{i}^{k}$ is the fused discrete correction estimate for station i in the kth unit, with units of time. $\Omega_{i}(k)$ is the set of valid reference stations connected to station i, $w_{ij}^{k}$ is the confidence weight of the corresponding station pair, and $\tau_{ij}^{k}$ denotes the station-pair residual after conversion into the correction direction of station i. A positive $\delta_{i}^{k}$ means that the station-i waveform is advanced by that amount in the later correction step.

To reduce the influence of anomalous observations on the fusion result, a second reweighting step updates the station-pair weights according to the observation residuals:

(12) $$w_{ij,rob}^{\left(k\right)}=w_{ij}^{\left(k\right)}\;\psi \left(\frac{\left|\tau_{ij}^{\left(k\right)}-\delta_{i}\left(k\right)\right|}{\sigma_{r}}\right)$$

In Equation (12), the normalized residual is formed from the difference between an individual station-pair observation and the first-round fused correction result; in the displayed notation this residual is $\tau_{ij}^{k}-\delta_{i}^{k}$. $\sigma_{r}$ is the residual scale parameter with units of time, which can be estimated from the dispersion of valid residuals, and ψ(·) is a dimensionless robust weighting function. The updated weight $w_{ij,rob}^{k}$ remains dimensionless and reduces the influence of anomalous station pairs or abnormal time windows.

Here, the robust weighting function ψ(·) is used to control the penalty strength for the normalized residual u, and is given by:

(13) $$\psi \left(u\right)=\left\{\begin{array}{ll} 1, & \left|u\right|\leq c,\\ \frac{c}{\left|u\right|}, & \left|u\right|>c, \end{array}\right.$$

In Equation (13), u is the non-negative normalized residual, commonly written as $|\tau_{ij}^{k}-\delta_{i}^{k}|/\sigma_{r}$, and c is the dimensionless robust-threshold constant. Observations with u≤c retain their original contribution, whereas observations with u>c are downweighted in inverse proportion to the normalized residual, thereby limiting the effect of outlying station pairs on the correction curve.

### 3.5. Construction of the Continuous Time-Correction Curve

The residual-correction quantity $\delta_{i}^{k}$ at discrete time instants cannot yet be used directly to correct the continuous record and therefore needs to be fitted as a continuous time-correction curve $\delta_{i}(t)$. In this study, the discrete residual-correction quantities are fitted with a cubic spline subject to a smoothing constraint, and obvious outliers are removed before fitting.

The objective of spline fitting is to balance the ability to track local variations with overall curve smoothness. If the smoothing constraint is too weak, the correction curve tends to follow local noise fluctuations; if it is too strong, it suppresses genuine residual-drift variations. For this reason, the smoothing term and the fitting term are jointly included in the optimization objective, and a suitable smoothing parameter is selected through preliminary experiments.

For long-duration records, a continuous time-correction curve has greater practical value than a discrete time-shift table. On the one hand, it can be used directly for resampling or timestamp correction; on the other hand, it can also serve as a diagnostic quantity of system operating status, thereby helping identify synchronization anomalies during specific periods.

From the perspective of discrete implementation, the spline-fitting problem can be written as a weighted least-squares problem with a second-difference regularization term:

(14) $$J_{i}\left[f_{i}\right]=\sum_{k=1}^{K_{i}}\rho_{i}\left(k\right){\left[\delta_{i}\left(k\right)-f_{i}\left(t_{k}\right)\right]}^{2}+\lambda \int {\left[f_{i}\prime \prime \left(t\right)\right]}^{2}dt$$

In Equation (14), $J_{i}(f_{i})$, equivalently $J(d_{i})$ in vector notation, is the objective function minimized for station i. The fitted curve $f_{i}(t)$ represents the continuous time-correction curve of station i; its values at the discrete time nodes form the vector $d_{i}$, with units of time. The observed fused corrections $\delta_{i}^{k}$ form the observation vector d^i obtained from the multi-station fusion step, and $\rho_{i}^{k}$ is the confidence weight of the kth observation, corresponding to the kth diagonal entry of the weight matrix W. The first term measures the weighted mismatch between the fitted curve and the observed residual corrections, whereas the second term penalizes rapid temporal fluctuations. The smoothing operator is implemented as a second-difference or curvature operator L in the discrete form, and λ is the smoothing regularization coefficient controlling the trade-off between data fitting and curve smoothness. A smaller λ allows the fitted curve to follow local residual variations more closely, whereas a larger λ produces a smoother correction curve. Both $d_{i}$ and d^i have units of time.

For convenience of implementation, the continuous time-correction curve can be represented by cubic-spline basis functions:

(15) $${\hat{\delta }}_{i}\left(t\right)=f_{i}\left(t\right)=\sum_{l=1}^{L}\beta_{i,l}\;B_{l}\left(t\right)$$

In Equation (15), $\delta_{i}(t)$ and $f_{i}(t)$ denote the same continuous station-specific time-correction curve for station i, measured in seconds or microseconds. $B_{l}(t)$ is the lth cubic-spline basis function, $\beta_{i,l}$ is its coefficient for station i, and L is the total number of basis functions. This spline representation transforms the discrete residual corrections into a continuous correction function that can be evaluated at arbitrary time samples.

After discretizing the smoothing-constrained optimization problem, the following normal equation is obtained:

(16) $$\left(G^{T}RG+\lambda L^{T}L\right)f_{i}=G^{T}Rd_{i}$$

In Equation (16), G is the design matrix obtained by evaluating the spline basis functions at the discrete time nodes, and it corresponds to A in the standard spline-coefficient notation. R is the diagonal matrix of observation weights and is equivalent to the weight matrix W; L is the second-difference smoothing matrix; $f_{i}$ is the unknown vector to be solved for the fitted correction curve, corresponding to the coefficient vector $a_{i}$ when the spline basis form is used; and the right-hand-side vector $d_{i}$ corresponds to the observed fused correction vector d^i. The solution gives the spline parameters or equivalent curve values used to reconstruct the continuous correction curve $\delta_{i}(t)$.

### 3.6. Waveform Correction

Final waveform correction is implemented by updating timestamps or using fractional-delay interpolation. When the residual timing difference is smaller than one sampling interval but already sufficient to affect phase consistency, fractional-delay interpolation is superior to integer-sample shifting. In this study, a Lagrange interpolation-based fractional-delay method is adopted to preserve phase continuity of high-frequency components. The wavefield-domain residual correction comprises four main computational stages: cross-correlation calculation, reference-function construction, timing-difference estimation, and spline fitting.

After the continuous time-correction function is determined, the final waveform correction is completed by applying a continuous shift to the original time axis:

(17) $$x_{i}^{corr}\left(t\right)=x_{i}\left(t+{\hat{\delta }}_{i}\left(t\right)\right)$$

In Equation (17), $x_{i}^{corr}(t)$ is the corrected waveform of station i, $x_{i}(t)$ is the original waveform, and $\delta_{i}(t)$ is the continuous correction function estimated for that station, with units of time. Because $\delta_{i}(t)$ can be smaller than one sampling interval, fractional-delay interpolation is used rather than integer-sample shifting. Under the adopted sign convention, a positive $\delta_{i}(t)$ evaluates the original trace at a later time sample and therefore advances a waveform that was delayed.

## 4. Case Study of Residual Timing Correction

To verify the effectiveness of the proposed method, three comparison conditions are adopted in the experiments: B0 denotes no residual correction and direct use of the original timestamps, B1 denotes hardware synchronization only, and B2 denotes hardware synchronization plus wavefield-domain residual correction. The method is evaluated from six aspects: long-term residual delay, joint timing and waveform-domain metrics, frequency-dependent phase error, parameter sensitivity, robustness, and application-level benefit.

The data consist of repeated calibration events, long-duration background records, and local passive-source samples. All methods are compared using the same set of raw records, the same analysis band, the same window length, and the same evaluation metrics to ensure comparability of the results.

### 4.1. Test Conditions and Methodology

#### 4.1.1. Case Data and Array Configuration

**Figure 1:**
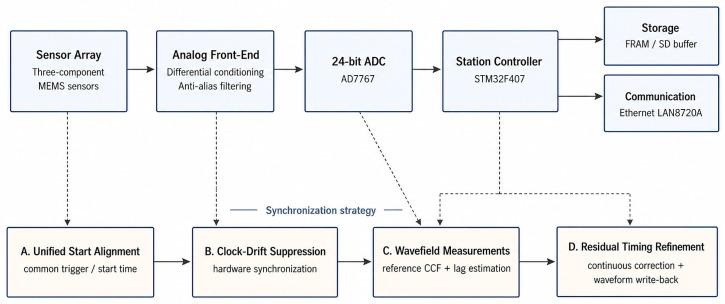
Architecture of the multi-source acoustic detector platform and its synchronization strategy.

The multi-source acoustic detector platform used in this case and its hardware components are shown in Figure 1. The platform provides unified start-up acquisition, continuous recording, buffering, and network transmission, and forms the data-acquisition basis for subsequent wavefield-domain residual-correction experiments. The system platform provides stable and repeatable data-acquisition conditions as well as a unified timestamp basis, whereas wavefield-domain residual correction is used to realize posterior fine refinement [10,11].

The experimental platform uses a local array composed of six acquisition stations with an inter-station spacing of 1 m and a nominal aperture on the order of 12 m, and each station is connected to 12 channels. The sample records are acquired at a sampling rate of 1000 Hz and each continuous record lasts 60 min. For calibration events, repeated hammer impacts are used as a repeatable wavefield excitation, and three groups of representative hammer-impact events are arranged within the intervals 20–30 min, 30–40 min, and 40–50 min, respectively. For background records, stable noise segments are selected to construct ambient-noise statistical windows.

First-arrival picking was performed using the same semi-automatic procedure for all comparison conditions. For each hammer-impact event, a candidate first arrival was first detected within the predefined event window from the short-time energy increase and the waveform-onset position, and the candidate picks were then manually reviewed only to remove visually obvious false triggers or low-quality picks. No manual adjustment was made separately for B0, B1, or B2, and the same picking window, threshold, and quality-control rule were used for all three conditions. Therefore, the reported arrival-time dispersion, repeated-pick standard deviation, and location-related metrics reflect the effect of residual timing correction rather than a change in the picking strategy.

#### 4.1.2. Baseline Settings and Comparison

The three conditions B0, B1, and B2 correspond to no residual correction, hardware synchronization only, and hardware synchronization combined with wavefield-domain residual correction, respectively. B0 is used to characterize the scale of synchronization error in the raw records, B1 to characterize the existing synchronization capability of the engineering system, and B2 to characterize the gain brought by the proposed method. The three conditions are evaluated using the same set of raw records to avoid spurious gains caused by data differences.

In the result analysis, the difference between B0 and B1 mainly reflects the error-convergence effect produced by hardware synchronization, whereas the difference between B1 and B2 characterizes the additional gain of wavefield-domain residual correction. This comparison strategy makes it possible to distinguish the actual contribution of residual correction from the synchronization capability already provided by the system.

#### 4.1.3. Evaluation Metrics and Statistical Procedures

Time-domain metrics include maximum inter-station deviation, root-mean-square jitter, residual delay at the final time, and mean absolute error. Waveform-domain metrics include the cross-correlation peak coefficient, peak timing difference, half-peak width, and arrival-time dispersion. Application-level metrics include location dispersion radius, stacking energy concentration, and the standard deviation of repeated picks.

For time-domain metrics, repeated experiments are taken as the statistical unit, and the mean, standard deviation, maximum value, and 95th percentile are reported. For waveform-domain metrics, station pairs or analysis windows are taken as the statistical unit, and the mean and fluctuation range are reported. Application-level metrics are uniformly compared within the same event family to ensure consistent statistics before and after correction.

To compare error-suppression performance and application-level benefit, three evaluation formulas are used: RMS jitter in Equation (18), arrival-time dispersion in Equation (19), and the relative gain index in Equation (20).

(18) $$RMS=\sqrt{\frac{1}{N}\sum_{n=1}^{N}e_{n}^{2}}$$

In Equation (18), RMS jitter is the root-mean-square value of the residual timing errors and has the same time unit as the residual-error samples. $e_{n}$ is the nth residual-error sample, and N is the total number of samples used in the statistic.

(19) $$\mathrm{Scatter}=\sqrt{\frac{1}{M}\sum_{m=1}^{M}{\left(t_{m}-\overset{-}{t}\right)}^{2}}$$

In Equation (19), Scatter denotes the arrival-time dispersion of repeated picks and is expressed in the same time unit as the arrival measurements. $t_{m}$ is the mth arrival-time measurement, $\overset{-}{t}$ is the mean arrival time, and M is the number of arrival-time measurements included in the calculation.

(20) $$Gain=\frac{{Metric}_{B1}-{Metric}_{B2}}{{Metric}_{B1}}\times 100\%$$

In Equation (20), Gain is the relative gain index used to quantify improvement from hardware synchronization only to the proposed residual-correction condition. ${Metric}_{B1}$ and ${Metric}_{B2}$ are the values of the same metric under conditions B1 and B2, respectively. For error-type metrics, a larger positive Gain indicates stronger error suppression; for non-error metrics such as correlation peak coefficient or energy concentration, the improvement direction is interpreted according to the physical meaning of the metric.

#### 4.1.4. Parameter Sensitivity

The parameter-sensitivity experiment examines the effects of analysis-window length, processing band, confidence threshold, and the smoothing parameter in Equation (14). The analysis-window length is set to 8, 12, 16, 20, 24, 28, and 32 s; the processing band is switched among 5–50 Hz, 5–80 Hz, and 10–80 Hz; the confidence threshold is varied from 0.55 to 0.75; and the smoothing parameter is tested at 0.01, 0.1, 1, 10, and 100. The evaluation indices include the cross-correlation peak coefficient, residual delay at the final time, retained proportion of valid windows, and normalized correction-curve roughness. In this way, the combined impact of parameter variation on estimation accuracy, result stability, and usable data volume can be evaluated simultaneously.

#### 4.1.5. Robustness Experiment Design

The robustness experiment includes four degraded scenarios: low signal-to-noise ratio, injection of local anomalous windows, shortened analysis windows, and reduction in the number of valid station pairs. The low-SNR condition is created by superposing controlled noise on the calibration events, anomalous-window injection is realized by artificially introducing impulsive interference, and the reduction in valid station pairs is achieved by removing some low-quality links. The purpose of the robustness experiment is not to pursue optimal results under extreme conditions, but to examine the degradation slope of the proposed method as observation quality decreases. If the method can still maintain low residual error under degraded conditions, its engineering applicability is demonstrated.

#### 4.1.6. Event-Location Validation Design

The significance of wavefield-domain residual correction lies not only in improving timing statistics, but also in whether such improvements can be transformed into more stable geophysical interpretation results. For the location validation, the clustering dispersion of source positions under different synchronization strategies is compared; for the stacking validation, the energy concentration and repeated-pick stability of the same event family under different correction conditions are compared.

### 4.2. Results

#### 4.2.1. Long-Term Residual Delay Suppression

**Figure 2:**
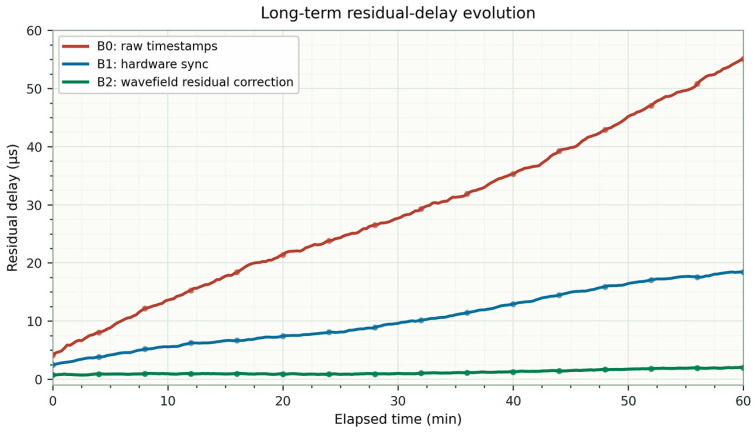
Evolution of residual delay over time under different synchronization strategies.

Figure 2 shows the evolution of residual delay over time under different methods. Under the no-correction condition, the residual delay reaches 55.1 μs at the end of 60 min. With hardware synchronization only, the residual delay at the final time is reduced to 18.4 μs, but a clear cumulative trend still remains. After wavefield-domain residual correction is introduced, the residual delay at the final time is further reduced to 2.0 μs. These results indicate that hardware synchronization can substantially reduce the error magnitude, whereas wavefield-domain residual correction removes the remaining fine-scale timing differences.

As shown in Table 1, the phase error at 50 Hz decreases from 6.6° under B1 to 0.7° under B2. This indicates that when high-frequency components are strong, residual timing errors can still significantly affect phase consistency even if hardware synchronization has already constrained the timing error to the order of several tens of microseconds. Therefore, wavefield-domain residual correction remains necessary.

#### 4.2.2. Characteristics of the Continuous Time-Correction Curves

**Figure 3:**
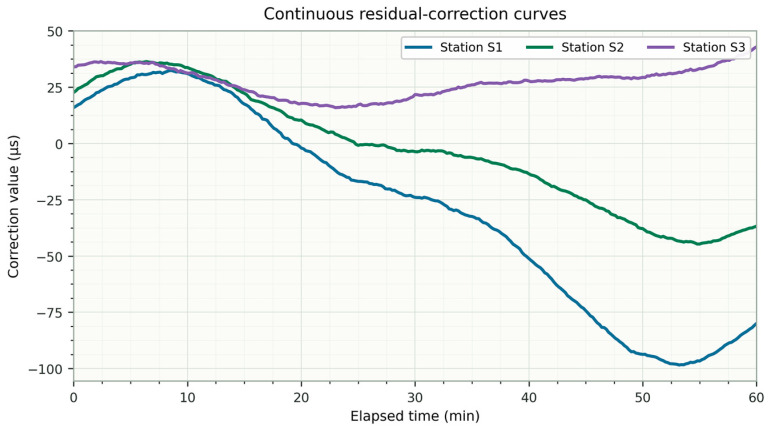
Continuous time-correction curves for representative stations.

Figure 3 shows the continuous time-correction curves for three representative stations. It can be seen that the residual-correction quantity does not vary identically with time for different stations: some stations exhibit slow monotonic drift, whereas others show low-amplitude fluctuations. This indicates that, in a local passive-source array, residual synchronization error is not determined entirely by a single global linear drift, but is more appropriately described by a smooth continuous function.

The overall variation range of the correction curves is less than 1 ms, but when applied to high-frequency waveforms it is sufficient to significantly affect phase and arrival time. If discrete time shifts are used directly without continuous fitting, unreasonable jumps may occur between adjacent time segments. After cubic-spline fitting, the correction curve becomes smoother and is also more suitable for direct resampling-based correction of long-duration records.

#### 4.2.3. Joint Timing and Waveform-Domain Metrics

**Figure 4:**
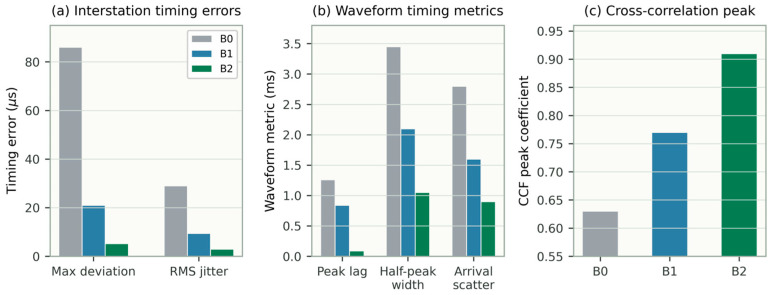
Multi-panel comparison of timing and waveform-domain metrics under different methods: (**a**) inter-station timing errors, (**b**) waveform timing metrics, and (**c**) cross-correlation peak coefficient.

Figure 4 and Table 2 present the joint timing and waveform-domain metrics using separated panels for quantities with different numerical ranges. Compared with hardware synchronization only, wavefield-domain residual correction reduces the maximum inter-station deviation from 21.0 to 5.2 μs and the RMS jitter from 9.4 to 2.9 μs. At the same time, the cross-correlation peak coefficient increases from 0.77 to 0.91, the peak timing difference decreases from 0.84 to 0.09 ms, and the arrival-time dispersion decreases from 1.6 to 0.9 ms. The split display prevents the large microsecond-scale timing-error values from masking smaller waveform-domain variations and shows that the proposed method improves both timing consistency and waveform coherence.

It is worth noting that the improvement in cross-correlation peak coefficient and arrival-time dispersion is smaller than that in maximum deviation and jitter, but their geophysical significance is more direct. The cross-correlation peak reflects the overall degree of waveform alignment, whereas arrival-time dispersion directly affects location and stacking stability. Therefore, these two metrics can be regarded as key evidence for the effectiveness of residual correction.

#### 4.2.4. Frequency-Response Analysis of Phase Error

**Figure 5:**
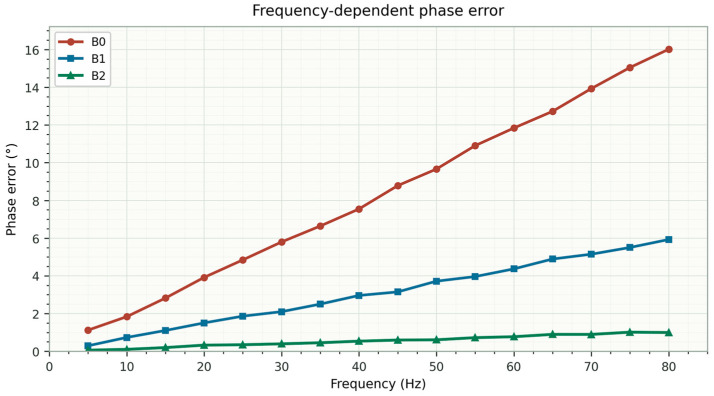
Variation in phase error with frequency.

Figure 5 shows the variation in phase error under different frequency conditions. Without residual correction, the phase error increases approximately linearly with frequency. After hardware synchronization only, the error slope decreases markedly. After wavefield-domain residual correction, the phase error remains within a small range below 80 Hz.

This result is consistent with the theoretical analysis of Equation (2); that is, for a given residual timing error, the phase error is proportional to frequency. Therefore, the benefit of residual correction becomes more pronounced in records containing richer high-frequency components. This feature is particularly important for near-field events, microseismic events, and short-duration high-frequency disturbances in passive-source seismic exploration.

#### 4.2.5. Parameter-Sensitivity Analysis

**Figure 6:**
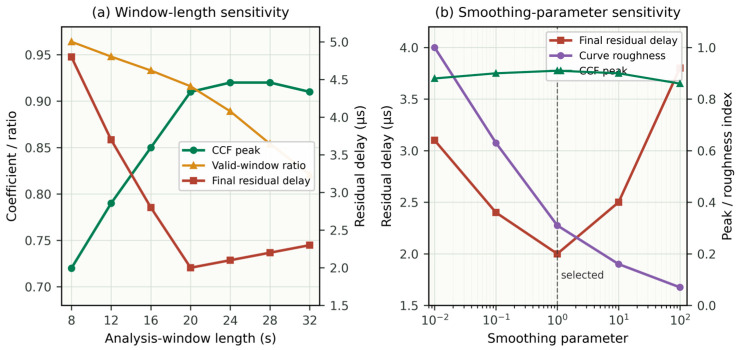
Sensitivity of the residual-correction result to (**a**) analysis-window length and (**b**) the smoothing parameter in Equation (14).

Figure 6 shows the sensitivity of the residual-correction result to analysis-window length and to the smoothing parameter in Equation (14). For the analysis-window length, the cross-correlation peak coefficient increases from 0.72 to 0.91 as the window length increases from 8 s to 20 s, while the residual delay at the final time decreases from 4.8 to 2.0 μs. As summarized separately in Table 3, further increasing the window length reduces the valid-window proportion without producing a clear additional reduction in the residual delay.

The smoothing-parameter test shows a similar trade-off between local fitting and temporal smoothness. When the smoothing parameter is too small, the fitted correction curve follows local fluctuations more strongly and the normalized curve-roughness index remains high. When the smoothing parameter is too large, the curve becomes overly smooth and cannot fully track genuine drift variations, which increases the final residual delay.

As Table 4 further shows, the minimum residual delay is obtained when the smoothing parameter is 1. At this value, the final residual delay is 2.0 μs, the cross-correlation peak coefficient is 0.91, and the normalized curve-roughness index is reduced to 0.31. In comparison, a smaller value of 0.01 gives a rougher correction curve and a final residual delay of 3.1 μs, whereas a larger value of 100 over-smooths the correction curve and increases the residual delay to 3.8 μs.

These results indicate that the selected analysis-window length of 20 s and smoothing-parameter value of 1 provide a practical compromise between estimation accuracy and correction-curve stability. Therefore, from the perspective of engineering deployment, the optimal parameter setting should not simply pursue the lowest single-point error, but should balance accuracy, robustness, time resolution, and curve smoothness.

Table 5 further evaluates the influence of frequency-band selection on the residual-correction result. The 5–50 Hz band retains more valid windows but excludes part of the high-frequency coherent energy, which lowers the CCF peak coefficient and leaves a larger final residual delay. The 10–80 Hz band suppresses low-frequency drift more strongly, but it also removes part of the stable low-frequency coherence and reduces the valid-window proportion. The 5–80 Hz band provides the best overall balance in this dataset, with a CCF peak coefficient of 0.91, a final residual delay of 2.0 μs, and a valid-window proportion of 91.6%; it is therefore used as the default processing band in the following experiments.

#### 4.2.6. Wavefield-Domain Cross-Correlation Enhancement and Robustness Analysis

**Figure 7:**
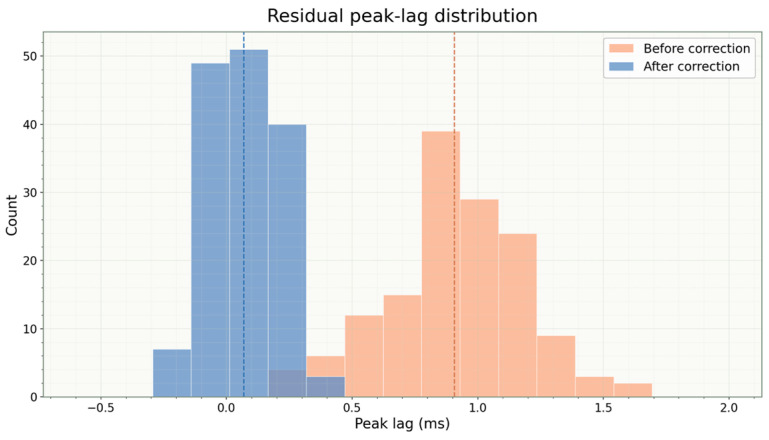
Distribution of peak timing differences before and after wavefield-domain residual correction.

Figure 7 shows the distribution of peak timing differences before and after residual correction. Before correction, the distribution is broad and offset from zero timing difference; after correction, the peak timing differences clearly converge toward zero and the distribution width is substantially reduced. This indicates that residual correction not only improves the mean timing offset, but also reduces the dispersion of overall wavefield alignment.

Table 6 presents the results under four degraded scenarios. Even under a low signal-to-noise ratio, shortened analysis windows, and the presence of anomalous windows, the proposed method still maintains relatively low residual error. In particular, under the low-SNR condition, the residual error under hardware synchronization only is 8.1 μs, whereas it decreases to 3.5 μs with the proposed method, demonstrating that confidence-based result screening and multi-station-pair fusion play a clear role in improving robustness.

Comparison among the degraded scenarios shows that the advantage of the proposed method does not lie in achieving exactly the same error level in all cases, but in maintaining a relatively gentle performance-degradation slope when observation conditions deteriorate. For example, when the number of valid station pairs is reduced by 30%, the residual error under B2 remains at 2.9 μs, indicating that multi-station-pair fusion retains sufficient redundancy even when some links are missing. When local anomalous windows are injected, the residual error under B2 is controlled within 4.2 μs, indicating that robust reweighting and outlier rejection directly suppress sudden anomalies.

The contraction of the distribution in Figure 7 also shows that the reduction in the variance of peak timing differences before and after correction is clearly larger than the reduction in the mean offset. For passive-source interpretation, this means that the benefit of correction is manifested mainly in reducing inconsistency among events or analysis windows, rather than only removing a fixed bias. It follows that the proposed method not only improves average time alignment, but also enhances record repeatability and stackability.

#### 4.2.7. Comparison of Effects on Event Location

**Figure 8:**
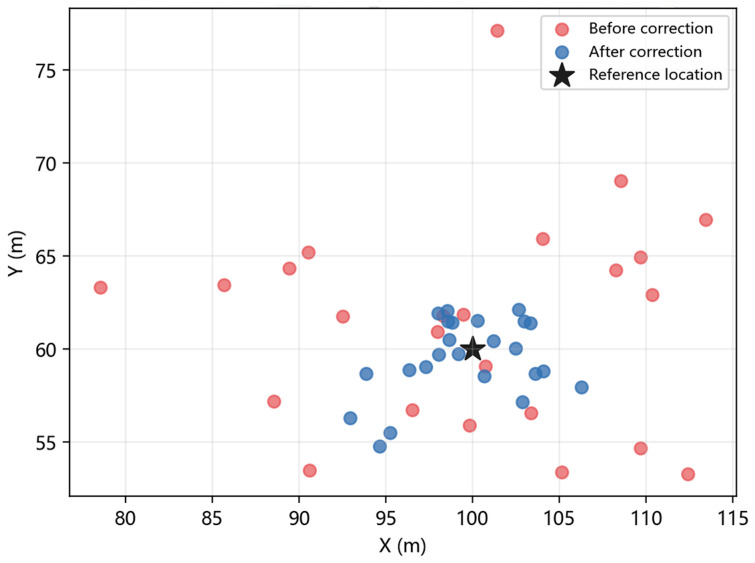
Comparison of source-location clustering before and after wavefield-domain residual correction.

Figure 8 and Table 7 give the event-location validation results. After wavefield-domain residual correction, the location dispersion radius is reduced from 13.9 to 5.4 m, the stacking energy concentration increases from 64.2% to 82.1%, and the standard deviation of repeated picks decreases from 1.7 to 0.8 ms. These results show that the improvements in temporal and waveform consistency brought by residual correction can be further translated into more stable interpretation results and can directly improve the interpretive reliability of passive-source data. For local-array monitoring, this means that the same platform can obtain more stable location and stacking results through posterior algorithms without increasing additional hardware cost.

Further calculation using the relative gain index shows that the location dispersion radius decreases by 61.2%, the standard deviation of repeated picks decreases by 52.9%, and the stacking energy concentration increases by 27.9%. The magnitudes of improvement of these three types of metrics are not identical, which is related to their different sensitivities to synchronization error. Location and repeated picking depend directly on arrival-time consistency and are therefore more sensitive to residual correction. Stacking energy, in contrast, is influenced not only by temporal consistency but also by amplitude variation and propagation-path differences, so its improvement is relatively moderate.

#### 4.2.8. Waveform Display and Stepwise Processing Effects

In addition to the statistical metrics and event-location results, direct waveform displays are provided below to visually show the influence of each key processing step on the raw record, event consistency, and stacking effect, thereby verifying the improvement at the waveform level.

**Figure 9:**
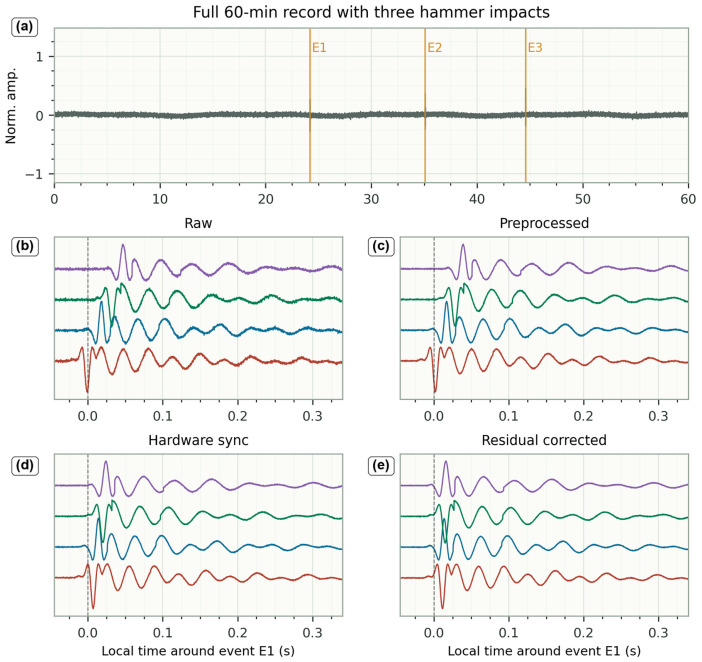
Stepwise waveform display for event E1. (**a**) Overview of the full 60 min record and the positions of the three hammer-impact events; the horizontal axis represents recording time in minutes. (**b**) Local raw multi-station waveforms around E1, (**c**) local waveforms after preprocessing only, (**d**) local waveforms after hardware synchronization, and (**e**) local waveforms after wavefield-domain residual timing correction. Colored traces in panels (**b**–**e**) distinguish different stations/channels; orange vertical lines in panel (**a**) mark E1–E3.

**Figure 10:**
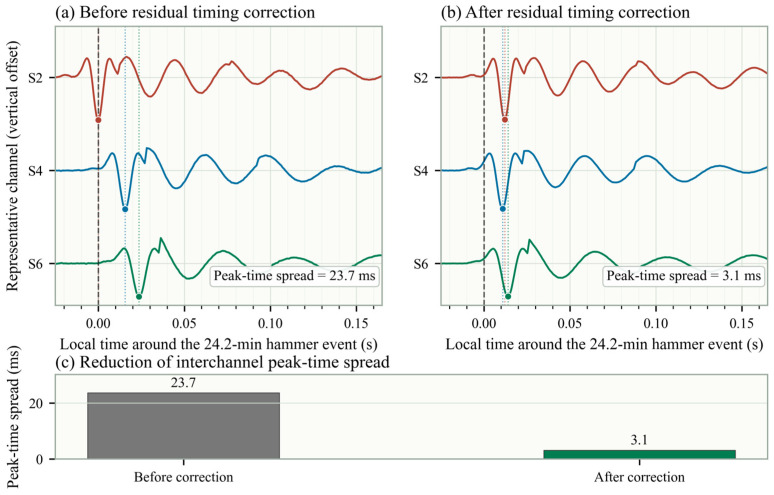
Waveform-level comparison for the 24.2 min hammer-impact event (E1) on representative channels S2, S4, and S6: (**a**) waveforms before residual timing correction, after hardware synchronization; (**b**) waveforms after wavefield-domain residual timing correction; and (**c**) reduction in interchannel peak-time spread computed from the strongest main-pulse peaks in the direct-arrival window. The traces in panels (**a**,**b**) are vertically offset; colored circles mark the selected main-pulse peaks, and the dashed black line marks the event-window reference time. Trace colors distinguish S2, S4, and S6, the peak markers use the same colors as their corresponding traces, and gray and green bars in panel (**c**) denote before and after correction, respectively.

**Figure 11:**
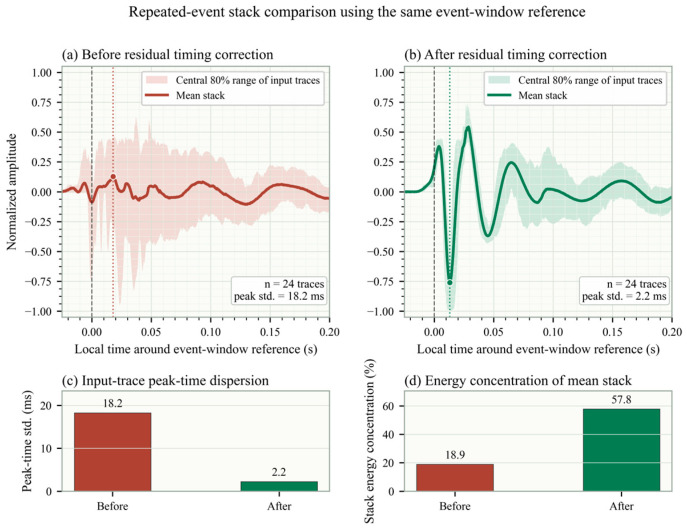
Repeated-event stacking comparison using E1–E3 and channels S1–S8: (**a**) mean stack before residual timing correction with the central 80% amplitude range of normalized input traces; (**b**) mean stack after wavefield-domain residual timing correction with the same display rule; (**c**) reduction in input-trace peak-time standard deviation; and (**d**) increase in stack energy concentration. The shaded band represents the central 80% amplitude range, i.e., the 10th–90th percentile range of normalized input traces at each time sample; the bold curve is the mean stack. Brown and green indicate before and after residual timing correction, respectively.

**Figure 12:**
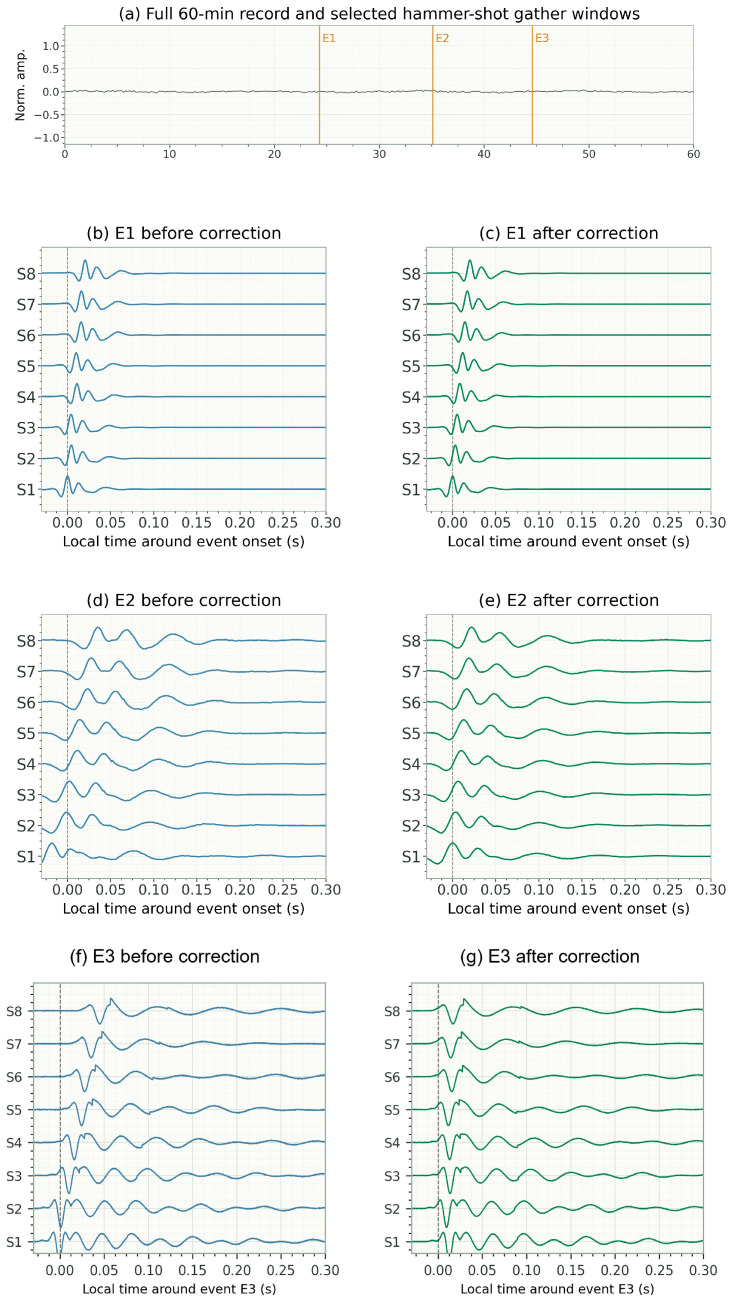
(**a**) Full 60 min record and the positions of the selected hammer-shot gather windows E1, E2, and E3; (**b**) local gather comparison of E1 before correction; (**c**) local gather comparison of E1 after correction; (**d**) local gather comparison of E2 before correction; (**e**) local gather comparison of E2 after correction; (**f**) local gather comparison of E3 before correction; and (**g**) local gather comparison of E3 after correction.

The waveform plots are organized to show the processing effect step by step. Figure 9 compares the same event window at four stages: the raw record, preprocessing, hardware synchronization, and wavefield-domain residual correction. Figure 10 gives a channel-level before-and-after comparison for one representative event, Figure 11 shows the corresponding change in stacked waveform sharpness, and Figure 12 extends the comparison to three representative event windows. This arrangement is intended to make the effect of each processing stage visually traceable rather than relying only on numerical indicators.

Figure 9 shows a 60 min continuous record and a locally enlarged view of the same hammer-impact event. Three representative events are visible in the full record. These three events were triggered artificially, which makes it possible to exclude the influence of other disturbance factors, and they are located in the time intervals 20–30 min, 30–40 min, and 40–50 min, respectively. The local enlargements compare the multi-station waveforms of the raw record, after preprocessing, after hardware synchronization, and after wavefield-domain residual correction. Visible misalignment of the main peaks exists among stations in the raw record; after preprocessing, the waveform envelopes become clearer; hardware synchronization narrows the overall timing-difference range; and after residual correction, the main peaks and subsequent oscillatory wave trains are further aligned.

Figure 9 is intended as a qualitative stepwise display rather than a velocity-estimation plot. Panel (a) provides the time context of the 60 min record, whereas waveform details should be read from the local panels (b)–(e). The similarity between panels (b) and (c) is expected because preprocessing suppresses abnormal amplitudes and unstable frequency components but does not align traces in time. Interchannel timing alignment is introduced only after residual-lag estimation and waveform time-axis correction, as shown by comparing panels (d) and (e).

Figure 10 compares the same 24.2 min hammer-impact event on channels S2, S4, and S6 before and after wavefield-domain residual timing correction. The three traces are vertically offset so that the main-pulse positions and coda-phase correspondence can be inspected directly. Before correction, the strongest main-pulse peaks of the three channels are more dispersed; after correction, these peaks become more concentrated in time.

The bottom panel gives a direct quantitative guide for reading the waveform panels. The interchannel peak-time spread decreases from 23.7 ms before residual timing correction to 3.1 ms after correction. This comparison is intended to validate the residual-lag estimation and waveform time-axis correction at the waveform level; it is not used to derive medium velocity, and the same event window and peak-selection rule are used before and after correction.

Figure 11 evaluates repeated-event stacking using the same event-window reference for E1–E3 and channels S1–S8. The same input set, normalization rule, event-window reference, and stacking rule are used before and after residual timing correction.

Before residual timing correction, the input traces have a broad peak-time distribution, and the resulting mean stack is weak and dispersed. After correction, the peak-time standard deviation decreases from 18.2 ms to 2.2 ms, while the stack energy concentration increases from 18.9% to 57.8%. No additional velocity model-based shift is applied in this display; the improvement reflects the residual timing correction and the same repeated-event stacking rule applied before and after correction.

Figure 12 further presents event-window gather comparisons for the three representative hammer-impact events E1, E2, and E3 distributed across the 60 min record. The newly added E1 and E2 windows show the same behavior as the original E3 window: before correction, local peak-position offsets and discontinuous main-energy bands are visible among stations, whereas after correction the main-energy bands become more continuous and the relative arrival-time relationships become more regular. The consistent improvement across the three representative events indicates that the correction effect is not an isolated result of a single event window, but a cross-event feature of the wavefield-domain residual timing correction.

Taken together, Figure 9, Figure 10, Figure 11 and Figure 12 show that the proposed method forms a complete waveform-level chain of evidence from preprocessing, residual timing-difference extraction, and multi-station fusion to continuous time-axis correction. The statistical improvements reported in the timing and waveform-domain metrics are therefore supported by direct multichannel evidence: the corrected traces show improved waveform alignment, more stable event repeatability, and more coherent gather continuity across E1, E2, and E3.

These displays also clarify the difference between preprocessing and residual timing correction. Preprocessing mainly improves waveform readability and cross-correlation usability by suppressing abnormal amplitudes and unstable frequency components, whereas residual timing correction changes the relative time alignment of coherent waveform features. The before-and-after plots therefore show not only that the traces become cleaner, but also that the main peaks, coda phases, and stacked-event energy become more consistently aligned after the correction workflow.

## 5. Conclusions

To address timing deviations that may still remain after hardware synchronization because of long-duration acquisition in passive-source seismic exploration, this study establishes a wavefield-domain residual-correction method. The method relies on waveform similarity in continuous records, uses the reference cross-correlation function as a comparison baseline, and combines residual timing-difference extraction, joint estimation from multiple station pairs, and continuous time-correction-function fitting to achieve post-acquisition correction of residual timing errors in long-duration records. The example results show that the method can reduce residual delay and phase error, improve cross-correlation peaks and arrival-time consistency, and improve location convergence and stacking performance. Overall, wavefield-domain residual correction can serve as an effective supplement to the synchronization-processing workflow of passive-source seismic exploration and can enhance the temporal consistency and interpretive stability of distributed acquisition data.

## Figures and Tables

**Table 1:** Long-term residual delay and phase error under different methods.

Method	Residual Delay at 60 min (μs)	Phase Error at 50 Hz (°)	Mean Absolute Error (μs)
B0: No residual correction	55.1	19.8	27.4
B1: Hardware synchronization only	18.4	6.6	7.2
B2 Hardware synchronization + wavefield-domain residual correction	2.0	0.7	1.1

**Table 2:** Joint timing and waveform-domain performance metrics.

Metric	B0: No Residual Correction	B1: Hardware Synchronization Only	B2: Proposed Method
Maximum inter-station deviation (μs)	86.0	21.0	5.2
RMS jitter (μs)	29.0	9.4	2.9
CCF peak coefficient	0.63	0.77	0.91
Peak timing difference (ms)	1.26	0.84	0.09
Half-peak width (ms)	3.45	2.10	1.05
Arrival-time dispersion (ms)	2.8	1.6	0.9

**Table 3:** Sensitivity results for analysis-window length.

Analysis-Window Length (s)	CCF Peak Coefficient	Final Residual Delay (μs)	Valid-Window Proportion (%)
8	0.72	4.8	96.4
12	0.79	3.7	94.8
16	0.85	2.8	93.3
20	0.91	2.0	91.6
24	0.92	2.1	88.9
28	0.92	2.2	85.4
32	0.91	2.3	82.1

**Table 4:** Sensitivity results for the smoothing parameter in Equation (14).

Smoothing Parameter	CCF Peak Coefficient	Final Residual Delay (μs)	Normalized Curve Roughness
0.01	0.88	3.1	1.00
0.1	0.90	2.4	0.63
1	0.91	2.0	0.31
10	0.90	2.5	0.16
100	0.86	3.8	0.07

**Table 5:** Sensitivity results for frequency-band selection.

Processing Band (Hz)	CCF Peak Coefficient	Final Residual Delay (μs)	Valid-Window Proportion (%)
5–50	0.86	2.8	94.7
5–80	0.91	2.0	91.6
10–80	0.84	3.2	86.9

**Table 6:** Robustness results under degraded scenarios.

Degraded Scenario	B1 Residual Error (μs)	B2 Residual Error (μs)	CCF Peak Coefficient
Low signal-to-noise ratio	8.1	3.5	0.84
Injection of local anomalous windows	9.7	4.2	0.82
Analysis window shortened to 10 s	6.4	3.1	0.80
30% reduction in valid station pairs	5.8	2.9	0.86

**Table 7:** Event-location validation results.

Application Metric	B1: Hardware Synchronization Only	B2: Proposed Method
Location dispersion radius (m)	13.9	5.4
Stacking energy concentration (%)	64.2	82.1
Standard deviation of repeated picks (ms)	1.7	0.8

## Data Availability

Data available on request from the authors.
